# Awareness about Breast Cancer and Breast Self-Examination among Undergraduate Female Students at the University of Agadir, Morocco: A Cross-Sectional Descriptive Study

**DOI:** 10.3390/epidemiologia5030028

**Published:** 2024-07-16

**Authors:** Malika Ben El-Fakir, Abdelmohcine Aimrane, Mehdi Ait Laaradia, Khalid Ait Taleb, Mohamed Omar Issaoune, Hasna Lahouaoui, Abdelaati El Khiat, Bilal El-Mansoury, Kholoud Kahime, Abdessamad Elmourid, Mohamed Ait-El-Mokhtar, Moulay Abdelmonaim El Hidan

**Affiliations:** 1 Laboratory of Biotechnologies and Natural Resources Valorisation, Faculty of Sciences, University Ibn Zohr, Ait Melloul 80000, Morocco; malikabenelfakir2015@gmail.com (M.B.E.-F.); khalid1.aittaleb@edu.uiz.ac.ma (K.A.T.); 2 Nutritional Physiopathologies, Neuroscience and Toxicology Team, Laboratory of Anthropogenic, Biotechnology and Health, Faculty of Sciences, Chouaib Doukkali University, El Jadida 24000, Morocco; el-mansouri.b@ucd.ac.ma; 3 Higher Institute of Nursing Professions and Health Techniques, Ministry of Health and Social Protection, Beni Mellal 23000, Morocco; mehdi.aitlaaradia@gmail.com; 4 Centre Hospitalier Universitaire Mohammed Vi Marrakech, Service d’Oncologie, Marrakech 40000, Morocco; issaoune.med.omar@gmail.com; 5 High Institute of Nursing Professions and Technical Health, Laâyoune 70000, Morocco; lahouaouihasna@gmail.com; 6 Biological and Health Sciences Team, Higher Institute of Nursing Professions and Health Techniques, Ministry of Health, Ouarzazate 45000, Morocco; abdelaatielkhiat@gmail.com; 7 SAEDD Laboratory, School of Technology Essaouira, Cadi Ayyad University of Marrakesh, Essaouira 44000, Morocco; kahimek@gmail.com; 8 Polyvalent Team in Research and Development (EPVRD), Department of Biology & Geology, Polydisciplinary Faculty, Sultan Moulay Slimane University, Beni Mellal 23030, Morocco; abdessamadelmourid1@gmail.com; 9 Laboratory of Biochemistry, Environment & Agri-Food URAC 36, Department of Biology, Faculty of Science and Techniques—Mohammedia, Hassan II University of Casablanca, Mohammedia 20000, Morocco; mohamed.aitelmokhtar@univh2c.ma

**Keywords:** breast cancer, awareness, breast self-examination, cancer risk factors

## Abstract

Breast cancer is a pressing public health issue globally and in Morocco, with rising cases among women. This study aims to evaluate breast cancer awareness and self-examination practices among female university students, informing future educational interventions. A cross-sectional study surveyed 437 students at Ibn Zohr University, Agadir, using a questionnaire covering demographics, knowledge of breast cancer, risk factors, symptoms, and breast self-examination (BSE). Results showed high awareness of breast cancer (95.3%), with social networks and media being primary information sources. However, only 48.25% had intermediate knowledge levels, and BSE awareness was moderate (60.8%) with low practical skills (28.0%). Reasons for not performing BSE included lack of knowledge and discomfort. Significant associations were found between knowledge levels and age, year of study, study options, and information sources. Despite high awareness, there is a crucial need to enhance knowledge about breast cancer risk factors, symptoms, and BSE practices among young women in Morocco. Educational programs targeting university students are essential for promoting early detection and improving attitudes toward breast health.

## 1. Introduction

Breast cancer is the most common cancer in women worldwide and the leading cause of cancer mortality in this population. According to the GLOBOCAN (2020), there are an estimated 2.3 million new cases [1]. Nearly 1 in 12 women will develop breast cancer in their lifetime. Breast cancer is the leading cause of cancer death in women, with nearly 685,000 dying from it up to 2020 [2]. The incidence rate is 47.8, and the mortality rate is 13.6 per 100,000 women, which imposes a considerable burden on public health [3]; the higher rate is observed in high-income countries compared to low and middle-income countries [4].

In Morocco, the age-standardized incidence of breast cancer was 47.5 per 100,000 women in 2017, according to the Casablanca Cancer Registry [5]. These statistics highlight the significant impact of breast cancer on women’s health in Morocco and emphasize the urgent need for effective prevention and treatment strategies.

Breast cancer is a complex and multifactorial disease that is attributed to both sporadic and familial factors. Indeed, less than 10% of breast cancers can be attributed to an inherited genetic mutation, particularly if they carry mutations in the BRCA1 or BRCA2 genes [6]. However, breast cancer is more commonly linked to environmental, mainly reproductive, and lifestyle factors (i.e., obesity, alcohol consumption) [7,8] in addition to hormonal influences, reproductive history [9,10], personal health history, age, gender, race, and breast density, collectively contributing to the multifaceted nature of breast cancer risk [11,12]. Furthermore, breast cancer represents various warning signs, including the appearance of a lump or mass, and skin alterations like redness, puckering, or dimpling [13], unknown pain, alterations in breast size or shape [14]. Early detection is crucial for successful treatment. Understanding these multifaceted risk factors and symptoms is crucial for individuals and healthcare professionals in devising effective prevention and screening strategies.

Routine self-examinations (BSE) are pivotal in the early detection of breast cancer [15,16], particularly in regions where access to regular medical check-ups and mammograms is limited, making self-examination even more critical [17]. However, disparities in breast cancer awareness and self-examination practices exist within the general population. Studies conducted in regions such as Egypt and the United Arab Emirates have highlighted inadequate awareness about breast cancer despite its prevalence, emphasizing the urgent need for improved education and awareness initiatives [18,19]. However, a study conducted among young females in Malaysia demonstrated a significant improvement in the practice of breast self-examination (BSE) following an intervention program [20]. Similarly, Korkut surveyed 668 women in Western Turkey, revealing that higher education levels were associated with increased frequencies of BSE [21]. Even among healthcare professionals in Morocco [22], Nigeria [23], and Rwanda [24], the variations in knowledge and practices are revealed, showing relatively superior awareness in physicians and nurses.

Among undergraduate students, research indicates varying levels of awareness and practices regarding breast self-examination (BSE). For instance, a study among 365 female undergraduate students in Nigeria revealed awareness of BSE, largely influenced by health workers. However, only 15.9% practiced BSE monthly. This study suggests increasing awareness through public health education and media campaigns, leveraging the influence of practitioners to encourage regular self-examination [25]. Similarly, Ahmed (2018) surveyed female students from six prestigious Karachi institutions to evaluate their knowledge, attitude, and practices regarding BSE. Their findings indicated that 71.4% of students were aware of BSE, with 33.1% having performed it previously [26]. A majority exhibited positive attitudes toward BSE, with a significant proportion rejecting traditional healing methods. Notably, a medical background was associated with higher levels of knowledge and positive attitudes [26].

In Morocco, research on knowledge, attitudes, and practices concerning breast cancer among female students is limited. Hence, the present study aims to assess breast cancer risk factors, warning signs, and the practice of breast self-examination among undergraduate females. By focusing on this demographic group, we seek insights into their knowledge, attitudes, and behaviors related to breast cancer awareness and BSE. We believe that understanding the perceptions and practices of these students could inform targeted educational interventions aimed at promoting breast cancer awareness and encouraging regular BSE practices, thereby contributing to the early detection and prevention of breast cancer in Morocco.

## 2. Materials and Methods

This study has a descriptive cross-sectional design. The study population comprised of all female students of the Ibn Zohr University campuses consists of three faculties: the Faculty of Applied Sciences; the Faculty of Languages, Arts, and Humanities; and the Faculty of Legal, Economic, and Social Sciences.

### 2.1. Sample Size and Sampling

The sample size was determined by Epi info software, with a precision of 5%, a confidence interval of 95%, and a 50% probability of having a high level of knowledge among students [27,28]. The calculated sample size required was 381.

This study included female university students at the three faculties of the university who expressed their willingness to participate in the survey. The exclusion criteria were incomplete responses. The final sample size was 429.

### 2.2. Study Instrument

A pretested, self-reported, semi-structured questionnaire was designed in both Arabic and French, the native and second languages used in the country, including informed consent, socio-demographic information, and questions related to knowledge of breast cancer, its risk factors, early symptoms, BSE practices, and its barriers, was prepared for this study through the extensive literature review [15,29].

The questionnaire comprised 34 items and was divided into six parts: participants’ socio-demographic characteristics (Section 1); general knowledge about breast cancer (Section 2); knowledge about and practice of BSE (Section 3); knowledge about breast cancer risk factors and symptoms (Section 4); barriers to early detection and diagnosis (Section 5); and perception about breast cancer treatment and its outcomes (Section 6).

### 2.3. Validation of the Questionnaire

To assess the reliability of the questionnaire, we used internal consistency and test–retest reliability measures, which indicated the level of repeatability of the scores over time. This estimation also reflects the stability of the characteristics evaluated by the test.

Nine participants completed the questionnaire twice at a three-week interval. The test–retest correlation showed good stability over time (intra-class correlation coefficient = 0.94); adequate internal consistency of the responses was observed with a Cronbach’s alpha coefficient of 0.73.

r = (n(Σϰy) − (Σx)(∑y))/√((nΣx^2 − (∑x)^2)(nΣy^2 − (∑y)^2)),

where r = 0.7336149933, given that n is the number of students; x is the first response, and y is the second response. The value of r is high. Therefore, we can conclude that the first-time responses are similar to those of the second time.

The questionnaire was reviewed by specialists before the pretest, and the results of the pretest were reviewed; the total question items retained were 34. The question elicited ‘yes’ or no’ or ‘true’ and ‘false’, and the correct answers were assigned one mark while the incorrect responses were assigned zero.

The level of breast cancer awareness was categorized as follows: (0–16 marks) low level; (17–24 marks) intermediate level; and (24–32 marks) high level. The maximum score was 32 marks.

### 2.4. Data Collection

This study was conducted over two months, from May to June 2021; verbal consent was obtained from students after explaining the confidentiality of the data obtained.

### 2.5. Data Analysis

Data were organized and coded using SPSS version 22 and Microsoft Office Excel; the statistical analysis included descriptive statistics, and Pearson’s chi-square test was used to determine the relation between categorical variables and breast cancer knowledge, BSE practice, and obstacles.

### 2.6. Ethical Consideration

Ethical approval to conduct this study was obtained from the University of Ibn Zohr Research Ethics Committee. The participants in the survey received an information sheet about the purpose of this study and were ensured that their participation would be anonymous and that their personal information would not be collected or exploited. All collected data were used only for research.

## 3. Results

### 3.1. Socio-Demographic Characteristics of Study Participants

In the current study, out of the total interview sample of 437 female students (Table 1), 429 were included in the analysis, and nine were excluded due to incompleteness, making the response rate 98.2%. The students’ mean age was 21.4 ± 2.8 (range 17–46 years). More than half of the students (n = 240; 56%) were aged 21–25. At the time of data collection, 189 (44%) students were in their third year (bachelor’s) of study at the university. In terms of study options, 167 (39%) of the respondents belonged to biological science, while the rest belonged to non-biological fields. Regarding the marital status of the respondents, the majority were single (n = 412; 96%).

### 3.2. Awareness of Breast Cancer and Sources of Information

Regarding the source of information, it was found that social networks played the most crucial role in spreading information (n = 150; 35%) among the candidates (Table 2). Other major sources of information chosen by the students were mass media (TV, radio) (n = 114; 27%), followed by 14.2% informed by peers and family members (46%, n = 11%). However, health facilities (n = 12; 3%) and other sources (n = 34; 8%) were found to have a limited role in breast cancer and breast self-examination awareness in the sample.

Results showed (Table 2) that all participants had heard of breast cancer. The majority of the respondents (92.1%) affirmed that breast cancer was not a rare disease and could not be transmitted from one person to another, as claimed by 95.1% of students. Moreover, most of the respondents (84.6%) declared that breast cancer was not less aggressive with advanced age, and 83.4% attributed the disease to old age. Most of the participants affirmed that breast cancer was curable (96.7%) and not contagious (93.7%). Furthermore, 87.4% affirmed that breast cancer was a serious disease, while 79.5% of them declared that breast cancer affected only women, and many of them believed the disease was not hereditary (69.5%).

### 3.3. Knowledge of Risk Factors of Breast Cancer Generally

More than half of the participants acknowledged breast trauma (63.6%), being a woman (61.1%), and smoking cigarettes (55%) as possible risk factors for breast cancer; however, the majority of the participants were unaware of complex risk factors such as early onset of menstruation (88%), birth of first child after the age of 30 years (80.2%), late menopause (73.2%), the family history (68.5%), the low-fat diet (64.8%) and the use of oral contraceptives (54.1%) (Table 3).

### 3.4. Knowledge of the Signs and Symptoms of Breast Cancer

As shown in the table below, the most commonly correctly identified sign of breast cancer was the presence of a breast lump or thickening in the breast (87.9%); the presence of mass or thickening in the armpit was the second most identified sign of breast cancer (77.2%), followed by the change in breast shape and size, nipple discharge, redness of the breast skin and painless breast lump in the following proportions: 72.7%; 72.3%; 71.3%; and 69.5%, respectively (Table 4). The nipple position change was not a well-known sign of breast cancer among the respondents (56%); however, the participants (90.2%) were successful in identifying that pain in the breast area is not a symptom of breast cancer.

The assessment of the students’ knowledge of breast cancer revealed that little less than half (n = 207; 48.3%) had an intermediate knowledge of breast cancer risk factors and their signs and symptoms (Table 5).

### 3.5. Awareness of Breast Self-Examination and Early Diagnosis

The scale for measuring students’ knowledge of BSEs consisted of six questions that were answered with either yes or no; the percentage of answers is presented in (Table 6). Results showed that 60.8% of the participants have heard of breast self-examination; however, 72% of the participants did not know how to practice breast self-examination, whereas, in terms of BSE practice, only 13.5% of the respondents in this study reported practicing or ever practiced breast self-examination. The overwhelming majority, 86.5%, reported not practicing BSE. Moreover, the majority of them (n = 425, 99.1%) claim that an early diagnosis is possible and effective, and more than half of the respondents (n = 257, 60%) affirm that mastectomy is not essential for curing breast cancer.

### 3.6. Perception of Breast Cancer and Breast Self-Examination and Treatments

The majority of participants in this study (89.5%) had correct beliefs about breast cancer management and its outcomes since they admitted that women enjoyed a good quality of life after a breast cancer treatment (Table 7). However, they had negative perceptions of breast cancer treatment by considering it to be a long-term and painful process (76.2%), while 76% believed that the treatments were more useful to young people. Most of the respondents (83.2%) claimed that the treatment was not embarrassing, and it did not lead to the loss of physical beauty, as affirmed by 60.4% of students; less than half of respondents (43.8%) thought that the breast cancer treatment was worse than the disease itself, and both radiotherapy and chemotherapy treatments were dangerous, according to 69.7% and 68.3% of all interviewed students, respectively.

### 3.7. Barriers to Early Diagnosis of Breast Cancer and Breast Self-Examination among the Studied Women

Table 8 presents the barrier predictors preventing the studied women from receiving an early diagnosis. According to the results, the most important obstacle was lack of knowledge (79. 3%); many respondents (76.7%) were worried about what a doctor might find; 60.1% of them found it difficult to touch their body by a stranger, while 59.7% felt embarrassed to reveal their breasts. More than half of the participants (59.2%) were scared of hospitals and care facilities, while 59% were afraid of mammography. On the other hand, most of the respondents (81.4%) affirmed no stigma associated with a cancer diagnosis; 64.3% had time to make an early diagnosis and did not feel embarrassed to tell people or even talk to a doctor, as said by 57.8% and 50.8%, respectively.

### 3.8. The Association between Breast Cancer Knowledge and Socio-Demographic Variables among Students

Age, marital status, year of the study, study options, and source of information were cross-tabulated against the knowledge score to determine their statistical significance. Out of the 47 respondents who had low knowledge, most of them 26 (55%) were aged between 21 and 25, and 18 (38%) were aged less than 20. Only three (6%) were aged above 26 (Table 9).

Among the 207 respondents who had intermediate knowledge, the majority were aged between 21 and 25, and among those who had high knowledge, more than half were aged between 21 and 25. There was a significant relationship between respondents’ ages and their levels of knowledge (*p* = 0.045).

The majority, 46 (98%), of the respondents who had poor knowledge were single, and only 1 (2%) was married. Also, among those who had intermediate knowledge, the majority, 199 (96%), were single, and 8 (4%) were married, while there were 167 (95%) participants who had high knowledge, and only 8 (5%) of them were married. 

There was no significant relationship between respondents’ knowledge and marital status (*p* = 0.7).

The majority (40.4%) of the respondents who reported poor knowledge attained a bachelor’s level (the third year), and (27.6%) had the first and the second year of a bachelor’s level. Only 4.2% of them attained master’s level (the fifth year). Also, among respondents that had intermediate knowledge, the majority (44.9%) of them attained a bachelor’s level (third year), and 23.67% attained the first year (bachelor’s level), while 5.3% had a master’s level (fifth year).

According to the high knowledge results, most of the participants (44%) were in their third year (bachelor’s level); 32% were in their second year; 16% were in their first year; furthermore, 6.3% of students were in their fourth year (master’s level); only 1.5% were in their fifth year (master’s level). There was a significant association between participant level of education and knowledge (*p* = 0.036). A total of 32% of students belonging to biology sciences, 25.5% to economic sciences, and 23.4% in the literature fields had poor knowledge about breast cancer. Among the respondents who had intermediate knowledge, 39% of them were in biology science (19%) and economic sciences, and 14% belonged to the literature options. However, of those who had high knowledge, 41.1% belonged to the biological field, 16% to economics, and 13.7% to the literature. There was a significant relationship between respondents’ study options and levels of knowledge (*p* = 0.039). Less than half (45%) of respondents with low knowledge of breast cancer are informed by social networks, 23.4% by media; regarding students with intermediate knowledge, 39% obtain information from social networks, and 25% from media. Of the participants with a high knowledge, 29% obtained information from the media, and 28% of them from the social network. Indeed, there was a significant relationship between an information source and level of knowledge (*p* = 0.03).

### 3.9. The Association between Breast Self-Examination (BSE) Knowledge and Socio-Demographic Variables among Students

Table 10 shows the relationship between BSE knowledge and socio-demographic characteristics; 57.5% of participants who have information about BSE are aged between 21 and 25, and the majority of them (95%) are single; 45.8% are in the third year (bachelor’s level), and 49.2% of the respondents with breast self-examination knowledge belong to the biology field and obtain their information from the social networks and the media, 31.7% and 25.8%, respectively.

For the breast self-examination knowledge and socio-demographic variables, it can be concluded that there is no significant relationship between such variables as age, marital status, year of the study, the study option, the information source, and breast self-examination knowledge since the *p*-value exceeds the critical value (*p* > 0.05).

## 4. Discussion

The current study is designed to explore the overall level of awareness about breast cancer, risk factors, early warning signs, and screening modalities, especially BSE, among university undergraduate students; 429 females out of the total interview questionnaires of a sample of 437 heard about breast cancer, making up the overall response rate 98.16%. This showed better awareness among young Moroccan females about breast cancer; these results align with Saba’s study. A total of 400 questionnaires were distributed, out of which only 381 were recovered, from who 373 heard of breast cancer (97%) [16], similar to the rate of 98.7% among female students at the University of Ibadan, Nigeria [17] and 95% among female university students in Ghana [30], and higher than the 88.1%, 81.2%, and 64% were observed in a group of Cameroonian [31], Malaysian [32], and Iranian [33], women respectively.

The students’ mean age was 21.4 ± 2.8 (range = 17–46 years). More than half of the students (n = 240; 56%) were aged 21–25; this age group belongs to reproductive age, and our findings corroborate with Godfrey’s study among female university students in Kampala, Uganda [29], and study by Gebresillassie, which was carried out in Northwest Ethiopia among female students of the university [34]. Other studies have documented average ages of 26.7 ± 1.9 years and 34.9 ± 12.2 years in the student population of Malaysia and in the general population of Saudi Arabia, respectively [35,36].

Regarding the marital status of the respondents, about 412 (96%) females out of a total of 429 were single, while 17 (4%) were married; our results are similar to the Ossai’s study, in which out of 365 female students, 94% were unmarried, while 6% were married [25]. This corresponds to the results obtained from most studies related to university students, where the percentage of single women is high [15,16,17,19,20,21,22,23,24,25,26,27,28,29,30,31,32,33,34,35,36,37].

At the time of data collection, 189 (44%) students were in their third year (bachelor’s level) of study at the university. In terms of study options, 167 (39%) of the respondents belonged to biological sciences, while the rest belonged to the non-biological fields.

Regarding the source of information, it was found that social networks played the most crucial role in spreading information (n = 150; 35%) among the candidates (Table 1). Other major sources of information chosen by the students were mass media (TV, radio) (27%; n = 114). Our finding is similar to the Egyptian [18] and Pakistan studies [16]; the widespread use of mobile technology may explain this. Also, our sample was a group of university students who tended to be savvy in modern technology, whereas a study among female Jordanian and Bangladeshi students reported healthcare providers and friends as the main source of their information [37,38]. It is, therefore, imperative to intensify efforts in using social media (advertisements, issues) to create breast cancer awareness and emphasize the importance of early detection, as this appears to be a better medium to reach a wider audience using various approaches.

This study shows a better awareness of breast cancer exhibited by the participants regarding the fact that breast cancer is a serious disease, not a rare disease, and cannot be transmitted from one person to another. Most of the respondents declare that breast cancer is not less aggressive with advanced age since the majority of respondents attribute the disease to old age.

Black women experience significantly higher breast cancer incidence up to the age of 40 and significantly lower incidence after the age of 50 compared with white women of the same age [39]. The overwhelming majority of participants affirm that breast cancer is curable, not contagious. However, a high percentage (79.5%) of them declare that breast cancer affects only women. This is higher than in an Ethiopian study conducted among 300 female university students, where 41% attributed breast cancer to females only [34]. In another study, 89% of the respondents of both sexes affirmed that breast cancer could affect men. Although it is rare, males can develop breast cancer [40].

Many of the respondents believed that the disease is not hereditary; other research yielded the same incorrect response, which showed that students were unaware that breast cancer was associated with genetic factors, which have a negative effect on breast health among young women. This low awareness of the role of genetic factors in breast cancer can be attributed to the low coverage of these risk factors in the media. In contrast, many studies show that a family history of breast cancer increases the risk of breast cancer [41,42].

The etiology of the majority of breast cancers is not known carefully, and only about 25% to 40% of them may be attributed to well-known risk factors [43]. The knowledge about breast cancer risk factors among university students is intermediate (48.5%); more than half of the participants acknowledged breast trauma as the first-factor risk (63%) and being women and cigarette smoking as possible risk factors for breast cancer. However, the majority of the participants were unaware of complex risk factors such as the early onset of menstruation, the birth of a first child after the age of 30, late menopause, and the use of oral contraceptives. Our result is similar to Godfrey’s study [29] and similar to a study carried out on students in Egypt to determine the awareness of breast cancer risk factors, where the findings also showed that very few students had knowledge of risk factors (late menopause, early menarche) as possible risk factors for breast cancer [44].

The participants failed to recognize the low-fat diet, which is similar to the Nigerian study, where less than 10% of teachers knew that obesity, early age of menstruation, and late age at menopause were breast cancer risks [15].

There have also been other reports regarding knowledge gaps regarding risk factors among the general population in Turkey and Egypt [45,46]. University students at the University of Sharjah in United Arab Emirates [19] and faculty from Najran University in Saudi Arabia [47], female medical students [48], female teachers [49], and female health care professionals and nurses [50].

Regarding warning symptoms, the most commonly correctly identified sign of breast cancer was the presence of a breast lump or thickening in the breast (87.9%). A similar study carried out in Uganda among women [51] revealed that the commonest warning sign of breast cancer, as cited by the participants, was a lump in the breast (60%), which was lower than the finding of our study, where presence of a mass or thickening in the armpit was the second most identified sign of breast cancer, followed by the change in breast shape and size, nipple discharge, redness of the breast skin, and painless breast lump. However, the nipple position change was not a well-known sign of breast cancer among the respondents (56%); the participants (90.2%) were successful in identifying that pain in the breast area was not a symptom of breast cancer.

The assessment of the students’ knowledge of breast cancer revealed that little less than half (48.3%) had an intermediate knowledge of breast cancer risk factors and their signs and symptoms, in contrast with Syed Azizur Rahman’s study among 241 female students at the University of Sharjah [19]. This suggests that the level of knowledge about warning signs and symptoms was insufficient (40%). The same results have been reported in an Indian study, which reported that less than 35% were aware of early signs of breast cancer [52].

Results showed that 60.8% of the participants had heard of breast self-examination; this finding corroborates with Ishtiak’s study, where three-fifths of the students interviewed heard about BSE [53]. This percentage is lower than in the Malaysian study, which was 97.1%. This is expected as the interviewers were all undergraduate health sciences students, and the programs included breast education and breast screening, including breast self-examination [54]. In general, health workers, nurses, and teachers showed a high level of BSE awareness. This reached 100% in the Nigerian studies [55,56,57].

However, only 28% of the participants know how to practice breast self-examination, although the majority of them (99.1%) claim that an early diagnosis is possible and effective. The awareness about breast self-examination has increased due to the increase in breast cancer rates all over the world, with the perception of breast cancer as a dangerous disease [57]. Surprisingly, in terms of BSE practice, only 13.5% of the respondents in this study reported regularly practicing or ever practicing breast self-examination. This is higher than the result mentioned in a cross-sectional study among 166 female university students in Cameroon that showed that only 3% of the students performed BSE regularly despite the fact that the awareness of BSE was 73.5% [31], with the lowest levels (1.3%) reported among female university students in Egypt [44], while the highest monthly practice level (78.3%) reported in Nigeria among female laboratory scientists [58]. Johnson’s review showed that though many of the studies reported a relatively high level of awareness of BSE in different countries in Africa, adequate practice was generally low [59]. Poor practice may be due to young women’s perceptions that they are healthy and, thus, do not need to perform a BSE. Moreover, the low practice could be attributed to a lack of knowledge about how to do BSE among young Moroccan students due to inadequate education programs about breast health awareness for this target population.

The barrier predictors prevent the studied women from receiving an early diagnosis. According to our results, the most important obstacle was the lack of knowledge. This aligns with other studies [60,61,62]. While many of the respondents were worried about what a doctor might find, more than half were scared of hospitals and care facilities; moreover, 60% were afraid of mammography. These psychological factors (fear and anxiety) are reported in Akhtari-Zavare’s study, which showed that among 810 female undergraduate students in Malaysia, 61.5% were scared of being diagnosed with breast cancer [63].

A fair percentage of respondents attribute not performing BSE to socio-cultural factors (shyness) since they found it difficult to have a stranger touch their bodies, and others felt embarrassed to reveal their breasts. This aligns with a previous Iranian study among women, where shyness, worries, and resistance against BSE related to fear are the most common barriers associated with breast self-examination [63].

Our results showed that respondents had positive attitudes toward BSE, as most of them affirmed that they had no stigma associated with a breast cancer diagnosis; they had time to make an early diagnosis, and they did not feel embarrassed to tell people or even talk to a doctor. This is consistent with the study from Palestine, where 38.1% of respondents did not feel embarrassed to do BSE, and 75.3% considered that BSE was not a waste of time [64].

The vast majority of research participants (89.5%) had accurate beliefs regarding the treatment of breast cancer and its consequences. They admit that women enjoy a good quality of life after breast cancer treatment. However, they had negative perceptions of breast cancer treatment by considering it to be a long-term and painful process (76.2%); this proportion is higher than in the Ethiopian study (42.3%) [34] and the Malaysian study (41.5%) [36].

Less than half of respondents (43.8%) said that the breast cancer treatment was worse than the disease itself; both radiotherapy and chemotherapy treatments are dangerous, according to 69.7% and 68.3% of the total interviewed students, respectively, while 76% believe that the treatments are more useful for young people, and most of the respondents 83.2% claim that the treatment is not embarrassing and does not lead to the loss of physical beauty, as affirmed by 60.4% of students.

- The association between breast cancer knowledge and socio-demographic variables among students.

Age, marital status, year of the study, study options, and source of information were cross-tabulated against the knowledge score to determine their statistical significance.

There was a significant relationship between respondents’ ages and levels of breast cancer knowledge (*p* = 0.04). According to the literature, the relationship between breast cancer knowledge and age is inconclusive. A study conducted in Ethiopia found that the overall level of knowledge on breast cancer was low among participants with a mean age of 21 [34]. Furthermore, young age has been identified as a strong risk factor for poor prognosis in breast cancer patients [54]. Another study conducted in the Akatsi South District of Ghana found that a large proportion of women surveyed were aware of breast cancer but had limited knowledge about its risk factors and symptoms, regardless of their age [65].

There was no significant relationship between respondents’ knowledge and marital status (*p* = 0.7), which is in contrast to a study conducted among students of Allied Health Sciences in Pakistan and Indonesia. Married students knew more about breast cancer than single or never-married students. This study suggests that being married or in a stable relationship may be associated with higher levels of breast cancer knowledge due to increased social support and access to health information [66]; furthermore, a study conducted in China reported that breast cancer awareness was associated with having a high level of health information literacy, husbands with higher education levels, etc. [67].

There was a significant association between participant level of education and breast cancer knowledge (*p* = 0.036). This aligns with Tayyeb’s study, where younger students in the Allied Health Sciences program scored worse on knowledge, while female students over 50 scored higher. Furthermore, postgraduate students showed more awareness of breast cancer markers and information than undergraduate students [66].

According to an Indian study, women with more than ten years of education are nearly four times more likely than women with less than ten years of education to be aware of breast cancer [68]. There was a significant relationship between respondents’ study options and levels of knowledge (*p* = 0.039). A similar observation was made in other studies of university students from United Arab Emirates and Angola [69,70]. The differences in knowledge score ratings between participants from different options and institutions may be related to each university’s varying courses or programs and their basic curriculums. Aside from this, basic breast cancer information should be taught to all young female students, regardless of their educational programs, to improve overall awareness and inspire beneficial breast cancer screening and preventative behaviors.

There was a significant relationship between information source and level of knowledge (*p* = 0.03). This is similar to another study that confirmed that mass media channels constitute the primary sources of information and could influence breast cancer knowledge since these sources of awareness were significant and effective in the practice of breast self-examination [71];

- The association between BSE knowledge and socio-demographic variables among students.

For the breast self-examination knowledge and socio-demographic variables, it can be concluded that there is no significant relationship between the following variables: age; marital status; year of the study; study options, information sources; and breast self-examination knowledge since the *p*-value exceeds the critical value (*p* > 0.05);

- The association between obstacle scores and socio-demographic variables among students.

There were no significant relationships between age, marital status, the year of study, the study options, the information source, and the obstacle score; the *p*-value exceeds 0.05.

## 5. Limitations of this Study

As the sample consists exclusively of female students, the results of this study can not be generalized to the larger population. Furthermore, as with any cross-sectional study, establishing causal relationships is challenging, and further research, including prospective methods, is warranted to overcome the potential study.

## 6. Conclusions

In this study, the overall level of knowledge on breast cancer was high in general, but the awareness about risk factors and warning signs was intermediate; the majority of the participants acknowledged old age and smoking as possible risk factors; however, the majority of the participants were unaware of complex risk factors such as family history, giving birth to the first child after the age of 30, early onset of menses, and menopause after the age of 50 years were linked to the risks of breast cancer. Pain in the breast region, change in the shape of the breast, and nipple discharge were the most frequently correctly identified symptoms of breast cancer. Even though participants had a positive perception toward breast cancer treatment and its outcomes and had a fair awareness of breast self-examination, a few of them performed it.

The age, the year of study, and the study option were found to be independently predicting factors for breast cancer knowledge among the participants, while this socio-demographic variable did not influence BSE knowledge.

## Figures and Tables

**Table 1:** Socio-demographic characteristics of the respondents and sources of information (n = 429).

Characteristics	Frequency (n = 429)	Percentage (%)
**Age (mean ± SD)**	21.4 ± 2.8	
≤20	162	38%
21–25	240	56%
≥26	27	6%
**Marital status**		
Single	412	96%
Married	17	4%
Year of Study		
Year 1 (Bachelor’s level)	90	21%
Year 2	116	27%
Year 3	189	44%
Year 4	20	5%
Year 5	14	3%
**Field of education**		
Biology Science	167	39%
Chemistry Science	41	10%
Physics Science	30	7%
Computer Science	18	4%
Economic Science	80	19%
Legal Science	29	7%
Letter	64	15%
**Source of information**		
A friend	73	17%
A family member	46	11%
A health facility	12	3%
Media	114	27%
Social network	150	35%
Other	34	8%

**Table 2:** Awareness of breast cancer among respondents (n = 429).

Variable	Frequency	
	Yes	No
Have you ever heard of breast cancer?	100% (n = 429)	0% (n = 0)
Breast cancer is a rare disease	7.9% (n = 34)	92.1% (n = 395)
Breast cancer affects only females	79.5% (n = 341)	20.5% (n = 88)
Breast cancer can be transmitted from one person to another	4.9% (n = 21)	95.1% (n = 408)
Old age	83.4% (n = 358)	16.6% (n = 71)
Less aggressive with advanced age	15.4% (n = 66)	84.6% (n = 363)
Breast cancer is curable	96.7% (n = 415)	3.3% (n = 14)
Breast cancer is a hereditary disease	30.5% (n = 131)	69.5% (n = 298)
Breast cancer is a contagious disease	6.3% (n = 27)	93.7% (n = 402)
Breast cancer is a serious disease	87.4% (n = 375)	12.6% (n = 54)

**Table 3:** Knowledge of risk factors of breast cancer by undergraduate students (n = 429).

Variables	Correct n (%)	Incorrect n (%)
Family history of breast cancer (yes)	31.5% (n = 135)	68.5% (n = 294)
Early onset of menstruation (before the age of 11) (yes)	11.7% (n = 50)	88.3% (n = 379)
Use of oral contraceptives (yes)	45.9% (n = 197)	54.1% (n = 232)
Breast trauma (yes)	63.6% (n = 273)	36.4% (n = 156)
Late menopause (yes)	26.8% (n = 115)	73.2% (n = 314)
Low-fat diet (yes)	35.2% (n = 151)	64.8% (n = 278)
Being a woman (yes)	61.1% (n = 262)	38.9% (n = 167)
Cigarette smoking (yes)	55% (n = 236)	45% (n = 193)
Birth of first child after the age of 30 years (yes)	19.8% (n = 85)	80.2% (n = 344)

**Table 4:** Knowledge of breast cancer symptoms by undergraduate students (n = 429).

Variables	Correct n (%)	Incorrect n (%)
Nipple retraction(yes)	59.2% (n = 254)	40.8% (n = 175)
Pain in breast region (no)	9.8% (n = 42)	90.2% (n = 387)
The appearance of wrinkles on the breasts (yes)	51.7% (n = 222)	48.3% (n = 207)
Nipple discharge (yes)	72.3% (n = 310)	27.7% (n = 119)
Presence of breast lump or thickening (yes)	87.9% (n = 377)	12.1% (n = 52)
Redness of the breast skin (yes)	71.3% (n = 306)	28.7% (n = 123)
Painless breast lump (yes)	69.5% (n = 298)	30.5% (n = 131)
Nipple position change (yes)	43.8% (n = 188)	56.2% (n = 241)
Presence of mass or thickening in the armpit (yes)	77.2% (n = 331)	22.8% (n = 98)
Change in breast shape and size (yes)	72.7% (n = 312)	27.3% (n = 117)

**Table 5:** Students’ level of knowledge of breast cancer, breast cancer risk factors, and symptoms (n = 429).

Characteristics	Frequency (n = 429)	Percentage (%)
Low (0–16)	47	10.9%
Intermediate (17–23)	207	48.2%
High (24–32)	175	40.8%

**Table 6:** Awareness of breast self-examination and early diagnosis among undergraduate students (n = 429).

Variable	Frequency	
	Yes	No
Have you ever heard of breast self-examination?	60.8% (n = 261)	39.2% (n = 168)
Do you know how to practice breast self-examination	28% (n = 120)	72% (n = 309)
Did you practice breast self-examination before?	13.5% (n = 58)	86.5% (n = 371)
Breast cancer can be prevented	92.1% (n = 395)	7.9% (n = 34)
Early diagnosis: possible and effective	99.1% (n = 425)	0.9% (n = 4)
Mastectomy is essential for curing breast cancer	40.1% (n = 172)	59.9% (n = 257)

**Table 7:** Perception about breast cancer and breast self-examination and treatments among respondents (n = 429).

Variable	Frequency	
	Yes	No
A woman, after treatment for breast cancer, can enjoy a good quality of life	89.5% (n = 384)	10.5% (n = 45)
Breast cancer treatment is a long and painful process	76.2% (n = 327)	23.8% (n = 102)
Breast cancer treatments are more useful to young people	76% (n = 326)	24% (n = 103)
Breast cancer treatment is embarrassing	16.8% (n = 72)	83.2% (n = 357)
Breast cancer treatment leads to loss of physical beauty	39.6% (n = 170)	60.4% (n = 259)
Treatment is worse than the disease itself	43.8% (n = 188)	56.2% (n = 241)
Radiotherapy is dangerous	69.7% (n = 299)	30.3% (n = 130)
Chemotherapy is dangerous	68.3% (n = 293)	31.7% (n = 136)

**Table 8:** Barriers to early diagnosis screening of breast cancer and breast self-examination among the studied women (n = 429).

Variable	Frequency	
	Yes	No
Possibility to touch my body	60.1% (n = 258)	39.9% (n = 171)
Embarrassing to tell people	42.2% (n = 181)	57.8% (n = 248)
Stigma associated with cancer diagnosis	18.6% (n = 80)	81.4% (n = 349)
I feel embarrassed to reveal my breasts	59.7% (n = 256)	40.3% (n = 173)
Scared of hospitals and healthcare facilities	59.2% (n = 254)	40.8% (n = 175)
Worried about what a doctor might find	76.7% (n = 329)	23.3% (n = 100)
Difficulty talking to the doctor	49.2% (n = 211)	50.8% (n = 218)
Lack of knowledge	79.3% (n = 340)	20.7% (n = 89)
Fear of doctors	51.7% (n = 222)	48.3% (n = 207)
Fear of mammography	59% (n = 253)	41% (n = 176)
Busy, no time for it	35.7% (n = 153)	64.3 (n = 276)

**Table 9:** Socio-demographic characteristics and knowledge of breast cancer.

Variables	Low Knowledge n = 47		Intermediate Knowledge n = 207		High Knowledge n = 175		Chi-Square Test
	Frequency	Percentage	Frequency	Percentage	Frequency	Percentage	
**Age**							
≤20	18	38.3%	78	38%	66	38%	0.04
21–25	26	55.3%	114	55%	100	57%	
≥26	3	6.4%	15	7%	9	5%	
**Marital status**							
Single	46	98%	199	96%	167	95%	0.7
Married	1	2%	8	4%	8	5%	
**Year of Study**							
Year I (Bachelor’s level)	13	27.6%	49	23.7%	28	16%	0.03
Year II (Bachelor’s level)	13	27.6%	47	22.7%	56	32%	
Year III (Bachelor’s level)	19	40.4%	93	44.9%	77	44%	
Year IV (Master’s level)	2	4.2%	7	3.4%	11	6.3%	
Year V (Master’s level)	0	0%	11	5.3%	3	1.7%	
**Study options**							
Biology science	15	32%	80	39%	72	41.1%	0.04
Chemistry science	2	4.2%	19	9%	20	11.4%	
Physics science	4	8.5%	12	6%	14	8%	
Computer science	2	4.2%	12	6%	4	2.3%	
Economic science	12	25.5%	40	19%	28	16%	
Legal sciences	1	2%	15	7%	13	7.4%	
Letter	11	23.4%	29	14%	24	13.7%	
**Source of information**							
A friend	9	19%	33	16%	31	18%	0.03
A family member	3	6.4%	19	9%	24	14%	
A health facility	1	2%	8	4%	3	2%	
Media	11	23.4%	52	25%	51	29%	
Social networks	21	45%	80	39%	49	28%	
Other	2	4.2%	15	7%	17	10%	

**Table 10:** Socio-demographic characteristics and breast self-examination practice knowledge.

Variables	Breast Self-Examination				Chi-Square Test
	**Yes**		**No**		
**Age**					
≤20	38	31.7%	124	40.1%	0.5
21–25	69	57.5%	171	55.3%	
≥26	13	10.8%	14	4.5%	
**Marital status**					
Single	114	95%	298	96.4%	0.5
Married	6	5%	11	3.6%	
**Year of Study**					
Year I (Bachelor’s level)	27	22.5%	63	20.4%	0.5
Year II (Bachelor’s level)	28	23.3%	88	28.5%	
Year III (Bachelor’s level)	55	45.8%	134	43.4%	
Year IV (Master’s level)	4	3.3%	16	5.2%	
Year V (Master’s level)	6	5%	8	2.6%	
**Study options**					
Biology science	59	49.2%	108	35%	0.08
Chemistry science	11	9.2%	30	9.7%	
Physics science	10	8.3%	20	6.5%	
Computer science	3	2.5%	15	4.9%	
Economic science	14	11.7%	66	21.4%	
Legal sciences	8	6.7%	21	6.8%	
Letter	15	12.5%	49	15.9%	
**Source of information**					
A friend	20	16.7%	53	17.2%	0.7
A family member	16	13.3%	30	9.7%	
A health facility	5	4.2%	7	2.3%	
Media	31	25.8%	83	26.9%	
Social networks	38	31.7%	112	36.2%	
Other	10	8.3%	24	7.8%	

## Data Availability

The original contributions presented in the study are included in the article, further inquiries can be directed to the corresponding authors.
